# Supplementary material for: A Rapid Realist Review of Group Psychological First Aid for Humanitarian Workers and Volunteers
Source: Int J Environ Res Public Health. 2021 Feb 4;18(4):1452. doi: 10.3390/ijerph18041452 (PMC7913971; doi:10.3390/ijerph18041452)
Supplement: Supplementary file 1 [file ijerph-18-01452-s001.pdf]

Table S1. CMOCs and PT Refinement

CMOCs Feeding into Programme Theory 1	Source
Context: Following a natural disaster in a low resource setting with limited mental health services access, GPFA was held within an appropriate time frame, in a safe venue away from the disaster area.<br>*Resource:* Mindfulness training and exercises were completed before and between sessions, and handouts were given to participants to support using, learning, and developing natural coping strategies.<br>*Reaction:* This helped participants normalise reactions and relationships, which<br>*Outcome:* can help to calm individuals initially and help them to manage overall stress reactions and prevent distress escalation.	[1]
Context: Following a natural disaster in a low-resource setting with limited mental health services access, GPFA was held within an appropriate time frame in safe venue away from the disaster area.<br>*Resource:* Participants were introduced to and taught about natural reactions to trauma and different coping strategies.<br>*Reaction:* This can help individuals to normalise their reactions and learn new coping strategies.<br>*Outcome:* Individuals are more empowered, better able to cope, have an 'I can do this' mentality. This helps to fosters long and short-term adaptive functioning and coping.	[2]
Context: Refugees were initially restricted to their camp when they first arrived, and are once again being restricted due to COVID-19. Past trauma is resurfacing in a low-resource setting, with many of the existing resources being cut off. PFA was provided by community workers,<br>*Resource:* provides psychoeducation about normal stress reactions and natural coping strategies.<br>*Reaction:* This can remind individuals of their previous coping strategies and supports their natural coping strategies.<br>*Outcome:* The outcome can be the prevention of distress escalation.	[3]
Context: During a conflict/war, with volunteers unsupported by the government, local volunteers have experienced similar trauma to those they are helping. When PFA training (led by an NGO) is held in a group setting,<br>*Resource:* the volunteers have the space and opportunity to connect and share with individuals going through similar experiences.<br>*Reaction:* Participants are able to learn from each other, which may<br>*Outcome:* increase feelings of social support and reduce feelings of isolation.	[4]
Context: Following a natural disaster in a low resource setting, when GPFA is held within an appropriate time frame,<br>*Resource:* individuals are provided psychosocial support and learn natural coping strategies such as mindfulness.<br>*Reaction:* Participants are equipped with the tools to process their experiences, which can<br>*Outcome:* help to prevent the escalation of distress and help survivors to cope and heal on their own.	[5]
Context: During an ongoing, stigmatising epidemic in a low resource setting with a collectivist culture, GPFA was held safely and as soon as was possible and appropriate.	[6]

Resource: Workers receive psychoeducation and support about normal reactions and positive coping strategies.<br>*Reaction:* Individuals have the tools and social support to process their experiences and reactions.<br>*Outcome:* The outcome can be the prevention of negative behaviour or coping strategies. This can help prevent the escalation of distress.	
Context: In a low-resource humanitarian crisis, volunteers tend to prioritise others before themselves and do not take time for self-care.<br>*Resource:* PFA/GPFA includes psychoeducation and teaches positive coping strategies that can be used every day. It can also include discussions challenging the notion that volunteers need to be brave and strong all of the time.<br>*Reaction:* Individuals are able to express their emotions/feelings to those who are going through similar experiences, and the normalisation in a group format can reduce hesitation about care-seeking among volunteers.<br>*Outcome:* Improved self-awareness and care, which can help to prevent distress from escalating.	[7]
Context: Following multiple disasters in a high-resource country, PFA was held as soon as possible and appropriate with survivors in shelters.<br>*Resource:* Individuals received psychoeducation and learned about the natural reactions to stress and trauma as well as positive coping strategies.<br>*Reaction:* Individuals are equipped with the tools needed to process their experiences, which can<br>*Outcome:* help to prevent the escalation of distress.	[8]
Context: Following staff witnessing trauma in a high-resource country, PFA should be provided as soon as possible and appropriate.<br>*Resource:* By receiving psychoeducation and learning positive coping strategies in a timely manner,<br>*Reaction:* members are able to process their experiences and reactions sooner.<br>*Outcome:* This can help to prevent escalation of distress over time.	[9]
Context: During conflicts that are ongoing or reoccur, some individuals have already received PFA previously.<br>*Resource:* Individuals are prepared and equipped with normalisation and coping strategies.<br>*Outcome:* This individuals may be able to remain calmer and more focused, and better able to protect themselves and those around them.	[10]
Context: Following an individual witnessing trauma in a high-resource country, when they<br>*Resource:* have someone to talk to about their emotions and concerns,<br>*Reaction:* individuals have increased feelings of belonging and support.<br>*Outcome:* This can help to decrease feelings of isolation, increase communication, and lead individuals to cope better on their own.	[10]

**CMOCs Feeding into Programme Theory 2**	**Source**
Context: Following a natural disaster in a high-resource country, not only does the disaster cause trauma, but also the period following the event if resource needs are disrupted, especially in an unfamiliar place. Evacuees needed basic needs met like food, a place to stay, clothes, support, medical care, etc.	[11]

Resource: There was a need for a system of complementary supports, for both physical and psychosocial needs.<br>*Reaction:* meeting these needs can an increase an individual's sense of stability, safety, and emotional expression.<br>*Outcome:* This can help to prevent the escalation of distress over time through reducing secondary stressors and helping individuals to cope on their own.	
Context: Following a natural disaster like a typhoon in a low-resource, collectivist country, people are often affected differently and may have various reactions to traumatic events. They may or may not have experienced something similar before. GPFA was provided in a safe location away from the disaster area, where participants felt comfortable.<br>*Resource:* Psychoeducation sessions about stress reactions and coping were provided to participants.<br>*Reaction:* This helped to promote self-efficacy, normalise reactions, and support emotional expression.<br>*Outcome:* The outcome is basic psychological needs are met, individuals are better able to cope on their own and prevent the escalation of distress.	[1]
Context: In the aftermath of a natural disaster in a low-resource, collectivist country, GPFA was provided in a safe location where participants felt comfortable.<br>*Reaction:* This helps to provide a sense of safety, which can<br>*Outcome:* help to prevent the escalation of distress over time through reducing secondary stressors.	[1]
Context: Following a natural disaster in a low-resource, low-income, collectivist society, individuals experience varying levels of stress, anxiety, and require different levels of support. When GPFA is provided in a safe location, away from the disaster area, where participants feel comfortable,<br>*Resource:* and provides information, psychoeducation, social, emotional, and psychological support in a group format,<br>*Reaction:* individuals feel an increased sense of safety and control over their situation, which can increase emotional expression.<br>*Outcome:* The outcome can be the stabilisation of emotionally distressed survivors, reducing secondary stressors, and preventing distress escalation.	[2]
Context: Volunteers in humanitarian crises tend to prioritise others before themselves, and do not take time for self-care. Volunteers are often affected differently and require different levels of support. When PFA/GPFA is provided, and it<br>*Resource:* provides coping strategies that can be used every day, and links to ongoing support and resources for individuals,<br>*Reaction:* individuals may be better able to express their emotions and needs to those who are going through similar experiences.<br>*Outcome:* The outcome can be the prevention of distress escalation and reduced secondary stressors.	[7]
Context: Following a sudden, violent event like a school shooting in a high resource country, people are affected differently	[10]

Resource: Group discussion provides practical information and support, assure safety, and provide psychoeducation about natural stress responses.<br>*Reaction:* Increases sense of safety and emotional expression<br>*Outcome:* Facilitates recovery from trauma, and prevents escalation of distress	

**CMOCs feeding into Programme Theory 3**	**Source**
Context: When people experience a similar traumatic event like a natural disaster, and GPFA is implemented early,<br>*Resource:* this can provide a space for activities and for people to express their emotions.<br>*Reaction:* Sharing is normalised, and survivors are more receptive about sharing their emotions.<br>*Outcome:* This can improve uptake and acceptability of GPFA.	[5]
Context: During an epidemic in a low-resource setting where mental health may sometimes be stigmatised, aid workers are affected differently and may require different levels of psychological support. When GPFA is provided,<br>*Resource:* it should facilitate service referrals and supports so that<br>*Reaction:* accessing services becomes normalised and accessible.<br>*Outcome:* Individuals will then be able to access the full psychological support that they need and that is right for them.	[6]
Context: In a humanitarian/refugee crisis in a low health resource country, people are often affected differently and require different levels of supports.<br>*Resource:* PFA should be provided by facilitators that are trained to work with various populations and age groups.<br>*Outcome:* This ensures that individuals are referred to and access additional services and care that is right for them and their current needs.	[12]
Context: Following a natural disaster in a high-resource country, basic resource needs may be disrupted, and people may be displaced. Individuals are affected differently and need different levels of support. When an existing support/resource system is already in place, PFA should be integrated and linked into that system.<br>*Resource:* Facilitators should be able to assess and identify needs, so that<br>*Reaction:* individuals can be referred to and access additional services and care that is right for them.<br>*Outcome:* This can reduce secondary stressors, which may help to restore some sense of normalcy/dignity to individuals.	[11]
Context: Following a natural disaster in a high-resource country, resources may be disrupted and some individuals may be displaced. When evacuees arrive to an unfamiliar place, they may face secondary stressors like finding a job, transportation, identification documents, etc. If there is an existing support/resource system in place, GPFA should be linked into that system.<br>*Resource:* Integration of psychosocial care with other resource services<br>*Reaction:* makes psychosocial support and psychoeducation more accessible.<br>*Outcome:* This can help to reduce secondary stressors while building a social support system. This can help individuals cope and prevent the escalation of distress.	[11]

Context: Following a natural disaster in a low-resource, collectivist country, people are affected differently and need different levels of support. [1]
Resource: Skilled facilitators gauge the levels of stress reactions among participants and identify those having more severe trauma reactions or symptoms. Those with significant symptom levels are grouped together and led by experienced counsellors.
Reaction: This ensure individuals receive the level of care and support that is right for them.
Outcome: The outcome is reduced secondary trauma to those around them, and respect for the individual's dignity and needs.

Context: Following a natural disaster in a low-resource, collectivist country, individuals are affected differently, have different responses, and may need different levels of support. [2]
Resource: When GPFA is provided, it creates an open space for members to share challenges, solutions, emotions, coping strategies, and resources.
Reaction: Individuals learn ideas from others and problem-solve together, and unmet or unrealised needs may be identified.
Outcome: The outcome can be reduced secondary stressors, and individuals are better able to cope on their own.

Context: Pandemics affect individuals differently, and individuals may need different levels of support. When GPFA is safely provided to refugees in camps, [3]
Resource: service and resource connections can be made for those who need more specialised care.
Reaction: This means that individuals are referred to and access level of care right for them and their current needs.
Outcome: The outcome can be that individuals are better able to cope on their own.

Context: Following a natural disaster in a low-resource, collectivist country, people react differently and require different levels of support. When PFA is provided [5]
Resource: and linked into the available services for those with more acute needs,
Outcome: individuals can be referred to and access additional services and the level of care right for them and their current needs.

Context: Following multiple natural disasters in a high-resource country, people have faced different levels of exposure, and are affected differently and require different levels of support. When PFA is [8]
Resource: provided by skilled facilitators able to gauge an individual's stress levels,
Reaction: high risk survivors can be identified and referred to more specialised care.
Outcome: The outcome is that individuals receive the level of care that is appropriate for them.

Context: Staff who witness trauma in a high-resource country are affected differently, in part due to their age or prior experiences, and may need different levels of support. When psychosocial support is provided [9]
Resource: with members who have previous experience coping with trauma,
Reaction: individuals can reflect on how they previously coped, and what worked and didn't work for them. This can be shared with other individuals experiencing trauma for the first time.
Outcome: Individuals are better able to prevent the escalation of distress and cope on their own.

Context: Following a sudden, violent event like a school shooting in a high-resource country with strong mental health support systems, people are affected differently and may require different levels of support.<br>*Resource:* When psychosocial services are provided and are tailored to individual needs,<br>*Outcome:* individuals can be referred to and access the level of care that is appropriate for them and their current needs.	[10]

**CMOCs feeding into Programme Theory 4**	**Source**
Context: Following a natural disaster in a high-resource country, service providers assisting displaced persons may be exposed to secondary trauma. If providers or team members were able get together to discuss their emotions or provide PFA to each other,<br>*Resource: c*ommunication may be fostered, and people may have felt comfortable sharing,<br>*Reaction:* which could increase social support and strengthen group cohesion.<br>*Outcome:* This may help to decrease feelings of isolation and enable providers to better help more disaster survivors.	[11]
Context: ECT staff had an ongoing, high risk of exposure to Ebola, witnessed traumatic scenes, and were concerned for safety of themselves and others. Staff were local to the communities they served, so many have witnessed suffering of those they knew. There was also loss of social connectedness because of the fear and stigma of the virus in the community. Cognitive behavioural therapy group interventions were led by peers to target depression and anxiety.<br>*Resource:* Workers have the opportunity to discuss their experience and fears with others going through the same event and can feel comfortable sharing.<br>*Reaction:* Participants have their feelings validated by others.<br>*Outcome:* This can help to decrease isolation and reduce distress	[13]
Context: ECT staff had an ongoing, high risk of exposure to Ebola, witnessed traumatic scenes, and were concerned for safety of themselves and others. Staff were local to the communities they served, so many have witnessed suffering of those they knew. There was also loss of social connectedness because of the fear and stigma of the virus in the community. Cognitive behavioural therapy group interventions were led by peers to target depression and anxiety.<br>*Resource:* Workers have the opportunity to discuss their experience and fears with others going through the same event and can feel comfortable sharing.<br>*Reaction:* This can strengthen group cohesion and sense of connectivity.<br>*Outcome:* This can help to decrease feelings of isolation and foster relationships and communication pathways.	[13]
Context: Community workers are refugees just like the communities to which they serve and have experienced the same or similar traumas. Refugee camps may have limited resources.<br>*Resource:* WhatsApp groups are promoted for community workers to have ongoing dialogue and to contact, check in, and provide space to talk with one another.<br>*Reaction:* Workers feel supported and a sense of belonging, which can<br>*Outcome:* help to reduce feelings of isolation and improve communication. Workers are then better able to support others.	[3]
Context: Volunteers working in humanitarian settings like refugee camps may face similar experience and stressors.	[7]

Resource: Volunteers are encouraged to establish a buddy system to look out and to support one another.<br>*Reaction:* This fosters accountability between workers, builds trust and feelings of support.<br>*Outcome:* This can lead to increased sense of safety, and decreased feelings of isolation, knowing that someone is looking out for them.	
Context: When people experience a similar traumatic event like sudden violence in a high resource setting (like a school shooting), and GPFA is provided,<br>*Resource:* provision of information and psychoeducation in a group format<br>*Reaction:* strengthens group cohesion and connectivity, people feel comfortable sharing, and can learn from each other.<br>*Outcome:* Group members feel less isolated and communication is fostered.	[10]
Context: When people are re-exposed to trauma in a low-resource setting, and GPFA is provided to a small group that already knows each other and shares a similar experience,<br>*Resource:* the group format<br>*Reaction:* may open communication about mental health within the group, strengthening group cohesion and sense of connectivity.<br>*Outcome:* Group members feel less isolated, communication and relationships are fostered.	
Context: Following a natural disaster like a hurricane in a high-resource country, which may result in displacement, evacuees may lose social support and be separated from loved ones. When group activities for these individuals take place,<br>*Resource:* this can promote community-centric activities<br>*Reaction:* which support a sense of belongingness.<br>*Outcome:* This can lead to decreased feelings of isolation, and the fostering of relationships and communication pathways.	[11]
Context: In a collectivist culture, when people who may not know each other experience a similar traumatic event, and GPFA groups are sorted into smaller groups of 5-7 members based on similar stress levels,<br>*Resource:* individuals are given the space to discuss their feelings and coping strategies.<br>*Reaction:* This can reinforce and strengthen an individual's sense of belongingness and strengthen group cohesion.<br>*Outcome:* This can help members to feel less isolated, build new relationships and communication pathways.	[1]
Context: In a collectivist culture, where many people already enjoy being in groups, when GPFA is provided to a small group where members may not know each other,<br>*Resource:* participants naturally feel comfortable sharing and expressing themselves in the group format.<br>*Reaction:* This supports solidarity, and individuals feel listened to.<br>*Outcome:* Individuals are more receptive and open to GPFA, as the group setting is aligned to the culture.	[2]

**CMOCs feeding into Programme Theory 5**	**Source**

Context: ECT staff faced an ongoing, high risk of exposure to Ebola and witnessed traumatic scenes. There was a loss of social connectedness because of the fear and stigma of the virus in the community. Mental health difficulties were often stigmatised in the communities. Group cogitative behavioural interventions were provided to heterogeneous groups of staff, led by peers to target depression and anxiety.<br>*Resource:* Members and facilitators were working at the same or similar levels.<br>*Reaction:* Staff were willing to attend the mental health interventions. Members felt comfortable sharing the challenges they faced & their coping strategies.<br>*Outcome:* ECT staff built social support within the team, learned new coping strategies, and reduced feelings of isolation and stigma associated with care seeking.	[13]
Context: When people experience a similar traumatic event, and GPFA is provided<br>*Resource:* to a whole family, or a heterogenous group who already knows each other,<br>*Reaction:* the group setting can mean more members are accessible and feel comfortable participating in and receiving PFA/GPFA.<br>*Outcome:* This can help to strengthen group cohesion and relationships, increase social support, reduce stigma associated with care seeking, and reduce feelings of isolation.	[14]
Context: When individuals from a collectivist culture experience a similar traumatic event like a natural disaster in a low-resource setting, and GPFA is provided by skilled facilitators to a<br>*Resource:* heterogeneous group made up of different ages, genders, life experience, and individuals do not know each other but are working at the same or similar levels,<br>*Reaction:* the group setting can help individuals feel comfortable sharing and expressing their emotions.<br>*Outcome:* This can help them to feel solidarity with other survivors, increase social support, and reduce feelings of isolation.	[2]
Context: Community workers are refugees just like the communities to which they serve, and have experienced the same or similar traumas. When support is provided<br>*Resource:* to a heterogenous group,<br>*Reaction:* the group setting can mean workers feel supported by both their peers and the organisation and can learn from each other.<br>*Outcome:* This may promote better communication, increase social support, and reduce feelings of isolation, which in turn enables them to better support those that they serve.	[3]
Context: When people in collectivist culturez experience a similar trauma like a natural disaster in a low-resource setting, and GPFA is provided to a<br>*Resource:* heterogeneous group working at the same or similar levels,<br>*Reaction:* the group setting can help build sense of companionship and support.<br>*Outcome:* This can promote social support and reduce feelings of isolation.	[5]
Context: During an ongoing epidemic, aid workers can become distressed from both their work and the community stigmatisation. When support is provided to a<br>*Resource:* heterogeneous group working at the same or similar levels,<br>*Reaction:* the group setting can mean members learn from each other and can feel comfortable sharing.	[6]

Outcome: This can help to reduce stigma associated with care seeking, promote social support, and reduce feelings of isolation.	
Context: Volunteers providing psychosocial support to refugees are often refugees themselves or have been through similar experiences to those they serve. When support is provided to a<br>*Resource:* heterogeneous group working at the same or similar levels,<br>*Reaction:* volunteers have a forum to share and learn from each other.<br>*Outcome:* The outcome can be stronger communication, promotion of social support and reduced feelings of isolation. This in turn encourages volunteers and workers to continue their work.	[7]
Context: When people witness trauma in a high resource setting and psychosocial support is provided<br>*Resource:* to a heterogeneous group working at the same or similar levels,<br>*Reaction:* members can feel comfortable sharing, discussing, and learning from each other.<br>*Outcome:* This can reduce stigma associated with care seeking, promote social support, and reduce feelings of isolation.	[9]
Context: When people experience a similar, sudden traumatic event like a school shooting, and GPFA is provided to a<br>*Resource:* heterogeneous group working at the same or similar levels,<br>*Reaction:* the group setting can help members to learn from each other, feel comfortable sharing, and strengthen group cohesion.<br>*Outcome:* This can help to reduce stigma associated with care seeking and reduce feelings of isolation.	[10]

**CMOCs feeding into Programme Theory 6**	**Source**
Context: Following a natural disaster that has resulted in displacement in a high-resource country, if<br>*Resource:* individuals are linked into existing community resources and wider support services<br>*Reaction:* this can help to build a social support system.<br>*Outcome:* This can help individuals be able to access social support, feel less isolated, and ensure that support is sustainable.	[11]
Context: Following a natural disaster that has resulted in displacement in a high-resource country,<br>*Resource:* when psychosocial support is built into existing mental health resources and linked into wider support services,<br>*Outcome:* more individuals are able to access support and support is more sustainable. This helps restore a sense of stability and reduce secondary stressors.	[11]
Context: Following a natural disaster that has resulted in displacement in a high-resource country, secondary stressors may lead individuals to not seek psychosocial or mental health support.<br>*Resource:* When psychosocial support is built on into existing resources and integrated into wider support services,<br>*Reaction:* more individuals are able to access psychosocial support in tandem with other resources.	[11]

Outcome: This can reduce stigma associated with care seeking, increase access, and ensure that the support is more sustainable.	
Context: When individuals are experiencing trauma during an epidemic that is stigmatised in a low resource setting where mental health is also stigmatised,<br>*Resource:* psychosocial support should be built into existing mental health resources when possible. (This intervention was not integrated into the local health system… 88% of citizens with mental difficulties seek traditional healers first… however a barrier here was that these healers were often in contact with sick patients & posed a high risk of contracting Ebola themselves.)<br>*Outcome:* This will help to ensure sustainability of support. (In this case, post-Ebola and post-intervention, there was not sustainable mental health support services)	[13]
Context: Following a natural disaster in a low resource setting, when<br>*Resource:* GPFA links individuals to services for practical assistance and social supports,<br>*Reaction:* awareness and visibility of mental health services and resources increases.<br>*Outcome:* This ensures that more individuals are able to access support and support is more sustainable.	[1]
Context: Following a humanitarian crisis or ongoing conflict in a low health resource setting,<br>*Resource:* PFA should be built on existing resources and integrated into wider support services.<br>*Outcome:* This enables more individuals to access ongoing support and ensures support is sustainable.	[14]
Context: Following natural disaster in low resource setting,<br>*Resource:* GPFA should be built into an existing network of support (in this case, it was not)<br>*Outcome:* This can help more individuals access support and ensure that support is sustainable. (Had GPFA been built on existing support… Those who were at risk or especially vulnerable could be referred to other services. Would have helped to ensure follow-through, link people with agencies, etc. and make the support overall more sustainable)	[2]
Context: When trauma resurfaces in a low resource, humanitarian setting, and PFA<br>*Resource:* is linked to existing resources,<br>*Reaction:* facilitators can refer individuals to resources and supports.<br>*Outcome:* This helps to ensure that individuals are able to access support and that support is more sustainable.	[3]

**CMOCs feeding into Programme Theory 7**	**Source**
Context: When people experience a similar traumatic event like a natural disaster in a low-resource setting, and GPFA is led by trained facilitators,<br>*Resource:* who are able to assess the current state of emotions/ well-being among survivors,<br>*Reaction:* facilitators are able to highlight the strengths of members, their needed support, etc.<br>*Outcome:* Individuals receive the care that they need, and there is reduced secondary trauma to other members.	[5]
Context: When the threat is ongoing (like Ebola) with safety fears, and group psychosocial support is led by peers targeting anxiety depression,	[13]

Resource: workers feel comfortable sharing and get the opportunity to discuss their experiences and fears with others going through the same event.
Reaction: Participants have their feelings validated by others, and get the opportunity to express and share,
Outcome: which can lead to decrease feelings of isolation and distress.

Context: When people experience a similar, sudden traumatic event like a school shooting in a high resource country, and GPFA is provided to a heterogeneous group made up of different ages gender or life experiences working at the same or similar levels, who already know each other, [10]
Resource: and are led by two trained facilitators,
Reaction: individuals with acute needs can be linked and referred to further services.
Outcome: This can reduce secondary trauma and ensure individuals receive the level of care that is right for them.

Context: In a humanitarian or refugee crisis in a low health resource country, people are often affected differently and require different levels of support. [12]
Resource: When GPFA is provided by facilitators that are trained to work with various populations and age groups, and linked into a layered system of complementary supports,
Outcome: Individuals can be referred to and access additional services and access a level of care that is right for them in their current needs.

Context: Following a natural disaster in a high resource country, when basic resource needs may have been disrupted and people may be displaced, individuals are affected differently in required different levels of support. When there is an existing support/resource system in place, [11]
Resource: and GPFA is integrated and linked into that system, and facilitators can identify and assess needs on arrival,
Reaction: individuals can be referred to and access additional services and care that is right for them.
Outcome: The outcome is that secondary stressors are reduced, restoring a sense of normalcy/dignity to individuals.

Context: Following a natural disaster in a low-resource, collectivist country, people are affected differently and require different levels of support. [1]
Resource: When GPFA is provided by skilled facilitators who can gauge levels of stress reactions and identify those who are having more severe trauma reactions or symptoms,
Reaction: this means individuals can receive a level of care that is right for them and their current needs and can be referred to the appropriate resources and services for them.
Outcome: The outcome is introduced secondary trauma to those around them as well as respecting their dignity/ individual needs.

Context: Pandemics effect people differently and people will require different levels of support. [3]
Resource: When GPFA is provided to refugees in camps by other community members that have been trained in PFA, at a safe distance,
Reaction: Individuals can be referred to and access additional services and care that is right for them and their current needs.
Outcome: The outcome can be reduced secondary stressors, and prevention of distress escalation.

Context: Following a sudden, violent event (like a school shooting) in a high-resource country, with strong mental health support systems, people are often affected differently and require different levels of support. [10]
Resource: when group PFA is provided by two facilitators per group, and links to further support, the second facilitator can monitor reactions of members, and take members aside who need further assistance.
Outcome: This means that individuals can be referred to and access additional services and access a level of care that is right for them and their current needs. This also helps to prevent members from being exposed to secondary trauma.

Context: Following multiple natural disasters in a high resource country, people have faced different levels of exposure and are often affected differently and require different levels of support. [8]
Resource: When GPFA is provided by skilled facilitators who can gauge individuals' levels of distress following the event,
Reaction: groups can be sorted based on levels of distress and individuals with acute or severe reactions can be referred to more specialised care.
Outcome: This means that individuals can be referred to and access additional services and access a level of care that is appropriate for them and their current needs. This also helps to prevent others from being exposed to secondary trauma.

References

1. Hechanova, M.R.M.; Ramos, P.A.P.; Waelde, L. Group-based mindfulness informed psychological first aid after Typhoon Haiyan. *Disaster Prevention and Management* **2015**, *24*, 610-618, doi:10.1108/DPM-01-2015-0015
2. Landoy, B.V.N.; Hechanova, M.R.M.; Ramos, P.A.P.; Kintanar, N.S.M. The Application and Adaptation of Psychological First Aid: The Filipino Psychologists' Experience After Typhoon Haiyan. *Psychological Association of the Phillipines* **2015**, *48*, 81-104.
3. Hussein Rasheed, R. Refugees deliver mental health services to locked down camps in Iraq. *ReliefWeb* 2020.
4. International Alert. Supporting the volunteers on front line of Ukraine conflict. International Alert: Ukraine, 2017.
5. Govt. Phillippines. Surigao quake survivors receive psych first aid, psychosocial support. ReliefWeb: Phillipines, 2017.
6. Mail & Gaurdian. Ebola burials traumatise aid workers. Mail & Gaurdian: Johannesburg, South Africa, 2015.
7. IFRC. *Coping with crisis - Focus: Volunteers - Capacity building*; IFRC: Copenhagen, Denmark, 2015; pp 1-24.
8. Shultz, J.M.; Kelly, F.; Forbes, D.; Verdeli, H.; Leon, G.R.; Rosen, A.; Neria, Y. Triple threat trauma: evidence-based mental health response for the 2011 Japan disaster. *Prehosp Disaster Med* **2011**, *26*, 141-145, doi:10.1017/S1049023X11006364.
9. Alisic, E.; Conroy, R.; Magyar, J.; Babl, F.E.; O'Donnell, M.L. Psychosocial care for seriously injured children and their families: A qualitative study among emergency department nurses and physicians. *Injury* **2014**, *45*, 1452-1458, doi:10.1016/j.injury.2014.02.015.
10. Turunen, T.; Haravuori, H.; Pihlajamäki, J.J.; Marttunen, M.; Punamäki, R.-L. Framework of the outreach after a school shooting and the students perceptions of the provided support. *European Journal of Psychotraumatology* **2014**, *5*, doi:10.3402/ejpt.v5.23079.
11. Legerski, J.-P.; Vernberg, E.M.; Noland, B.J. A qualitative analysis of barriers, challenges, and successes in meeting the needs of Hurricane Katrina evacuee families. *Community Ment Health J* **2012**, *48*, 729-740, doi:10.1007/s10597-011-9446-1.
12. Akoury-Dirani, L.; Sahakian, T.S.; Hassan, F.Y.; Hajjar, R.V.; El Asmar, K. Psychological first aid training for Lebanese field workers in the emergency context of the Syrian refugees in Lebanon. *Psychol Trauma* **2015**, *7*, 533-538, doi:10.1037/tra0000028.
13. Waterman, S.; Hunter, E.C.M.; Cole, C.L.; Evans, L.J.; Greenberg, N.; Rubin, G.J.; Beck, A. Training peers to treat Ebola centre workers with anxiety and depression in Sierra Leone. *Int J Soc Psychiatry* **2018**, *64*, 156-165, doi:10.1177/0020764017752021.
14. Schafer, A.; Snider, L.; Sammour, R. A reflective learning report about the implementation and impacts of Psychological First Aid (PFA) in Gaza. *Disaster Health* **2016**, *3*, 1-10, doi:10.1080/21665044.2015.1110292.
